# Moving towards transformative justice for black women survivors of intimate partner violence: an intersectional qualitative study

**DOI:** 10.1186/s12889-024-20244-y

**Published:** 2024-10-08

**Authors:** Laurel Sharpless, Trace Kershaw, Deja Knight, Julia K. Campbell, Karlye Phillips, Marina Katague, Tiara C. Willie

**Affiliations:** 1 Department of Mental Health, Johns Hopkins Bloomberg School of Public Health, 624 N. Broadway, 8th Floor, Baltimore, MD 21205 USA; 2 Department of Health Behavior, UNC Gillings School of Global Public Health, 135 Dauer Drive, Chapel Hill, NC 27599 USA; 3 Department of Social and Behavioral Sciences, Yale School of Public Health, 60 College Street, New Haven, CT 06510 USA; 4 Department of International Health, Johns Hopkins Bloomberg School of Public Health, 615 N. Wolfe Street, Baltimore, MD 21205 USA; 5 Department of Medicine, Division of Infectious Diseases and Global Public Health, University of California, San Diego, 9500 Gilman Drive, La Jolla, CA 92093 USA

**Keywords:** Intimate partner violence, Black women, Intersectionality, Transformative justice, Community

## Abstract

**Background:**

Intimate partner violence (IPV) disproportionately affects Black women, yet the current IPV justice response, relying on the criminal legal system, often fails them due to racialized, sexist stereotypes that disrupt Black women’s claims to survivorship. Transformative justice, a community-based approach designed to repair harm between the survivor and person who caused harm and transform the social conditions that perpetuate violence, may be a promising alternative approach to facilitate justice and accountability for IPV. However, little is known about the justice preferences of Black women IPV survivors. This qualitative study sought to understand Black women IPV survivors’ experiences interacting with police and their justice preferences following IPV.

**Methods:**

Semi-structured interviews with 15 Black women IPV survivors were conducted between April 2020 and April 2022. Inductive analytic techniques derived from grounded theory were used to contextualize Black women IPV survivors’ experiences.

**Results:**

One theme was identified that aligned with Black women IPV survivors’ experiences interacting with the police: 1) fear and distrust. Four themes were identified that aligned with justice preferences: (1) resolution through dialogue, (2) therapy and counseling services, (3) resource support, and (4) protection and prevention for children. Fear and distrust of the police was mainly driven by anticipated discrimination. Survivors’ justice preferences encompassed solution-based dialogue between the survivor and person who caused harm mediated by family and trusted individuals in the community, therapy services, housing support, and attention to preventing the intergenerational cycle of IPV for children as part of a community-based, holistic justice response.

**Conclusions:**

Police interactions as part of the current justice response were counterproductive for Black women IPV survivors. Black women IPV survivors deserve alternative forms of justice and accountability for IPV. As an alternative justice response to IPV, transformative justice can encompass their justice preferences and promote equity and center Black women IPV survivors and their communities.

**Supplementary Information:**

The online version contains supplementary material available at 10.1186/s12889-024-20244-y.

## Background

Intimate partner violence (IPV) is a prolific public health problem in the United States. IPV includes physical, sexual, psychological, and economic abuse by a current or former intimate partner or spouse [1]. IPV affects 1 in 3 women during their lifetime [2] and has a disproportionate impact on Black women. Specifically, 45.1% of non-Hispanic, Black women report experiencing lifetime IPV compared to 37.3% of non-Hispanic, white women [3]. IPV can result in multiple acute and chronic mental, physical, and sexual health consequences [4], and Black women IPV survivors are especially susceptible to adverse IPV-related health outcomes [5, 6].

### An intersectional approach to the primary justice response to IPV

Currently, the United States primarily relies on the criminal legal system as the primary justice and accountability response to IPV, which can be counterproductive for Black women IPV survivors [7]. The ineffectiveness of the criminal legal system for Black women IPV survivors can be traced back to the impacts of slavery and the nation’s devotion to white supremacy and patriarchy [8, 9]. Historically, Black women who experienced abuse from men, especially white men, were not protected and often devalued by the criminal legal system unlike their white women counterparts. For example, rape laws were only enforced when white women were victimized, which excluded Black women from receiving the same legal protection [9]. Additionally, Black women faced criminalization for self-defense against abusive men, often receiving harsher punishment than white women [8]. Currently, police act as gatekeepers of the criminal legal system, who Black women IPV survivors are supposed to turn to for help for IPV. However, police’s historical and current treatment of Black women IPV survivors reflects the longstanding oppression Black women faced by the criminal legal system during times of slavery [10].

An intersectionality theoretical framework can be used to understand and inform the historical and current criminal legal system response to IPV among Black women survivors. Intersectionality illustrates how social identities, such as race, sex, and class intersect at the individual level to give rise to experiences that embody interlocking systems of power and oppression, such as racism, sexism, and classism at a structural level [11–13]. These identities have a multiplicative effect and cannot, nor should not, be disentangled [14]. Black women cannot separate their identities of being Black and being a woman. When these two marginalized identities intersect, they face distinct structural challenges that differ from their white women or Black men counterparts [11].

Aligning with an intersectional lens, Black women IPV survivors face compounded structural inequities by the criminal legal system that are based on racist, sexist stereotypes that can result in barriers to achieving justice. For example, police officers often exhibit discriminatory, brutal, and disenfranchising behavior towards Black women who seek help for IPV [9, 15]. Compared to white women, Black women are more likely to be incarcerated during reports of IPV, particularly if they defend themselves during abusive events [8]. This treatment stems from stereotypes that label Black women as aggressive, portraying them as domineering and invulnerable, which delegitimizes their claims to survivorship [16–19].These perceptions contribute to the belief that a Black woman IPV survivor provoked her abuser, thereby criminalizing abuse victimization and sympathizing with abuse perpetration [20]. These stereotypes are also thought to contribute to the disproportionately higher rate of Child Protective Services (CPS) involvement that occurs when Black women IPV survivors report IPV to the police compared to white women IPV survivors [18, 21]. Additionally, Black women IPV survivors are more likely to lose custody of their children once CPS becomes involved than white women IPV survivors [22]. These negative experiences with ostensibly helpful social and legal service agencies often serve to further traumatize Black women IPV survivors and fail to promote safety and healing [18]. Taken together, this evidence suggests that Black women are disproportionately affected by both interpersonal and state violence, and these exacerbating forces produce an oppressive, inequitable justice response that often results in Black women’s apprehension to seek help for IPV, and consequently, a lack of justice served [9, 18].

### Transformative justice as an alternative approach to addressing IPV

Juxtaposing the workings of the criminal legal system, transformative justice has emerged as an alternative, holistic community-based justice approach to IPV prevention and intervention. Transformative justice was created, and is largely led by, Black women and other women of color [15]. In alignment with intersectionality’s commitment to transformation to advance equity and social justice [23], transformative justice embodies an abolitionist framework, and was created to provide a flexible, survivor-centered and solution-based justice and accountability response to IPV that centers marginalized communities, resists oppressive systems, and functions completely outside of the criminal legal system [24–26]. The intention behind transformative justice is to redesign the mechanisms that repair harm between a survivor and the person who caused harm without reinforcing the cis-heteropatriarchal racist, sexist, and classist structures of the criminal legal system [15]. Transformative justice recognizes that IPV occurs within a system of broader state violence, so addressing individual-level violent behavior alone (i.e., ignoring the structures that teach and perpetuate violence) is insufficient for IPV prevention and intervention [25].

The practice of applying transformative justice to IPV involves bringing the survivor and the person who caused harm together in a secure environment and centering the promotion of survivor agency and community change. Through this approach, the IPV survivor describes their narrative of harm they experienced, and the person who caused harm is encouraged to acknowledge the survivor’s experience, sincerely apologize, and work through rehabilitation [24, 26]. Transformative justice relies on members of the community to hold people who caused harm accountable and facilitate healing by recognizing that IPV occurs within the same neighborhoods that people who cause harm occupy. This can position the community to care about IPV resolution and the prevention of further violence [27]. Overall, transformative justice seeks to transform the survivor and person who caused harm and also seeks a process of transformation of the underlying community and systemic conditions that contribute to violence [15, 24]. For example, transformative justice envisions creating strategies to improve housing and education systems to contribute to the creation of safe communities, thereby removing the need to rely on the criminal legal system to address harm after it has occurred. Instead, the emphasis is on preventing and responding to harm by reducing adversities in a community’s systems that have the potential to teach and perpetuate it [25]. Given the barriers and inequities that Black women IPV survivors face when seeking justice from the criminal legal system, alternative responses to IPV for Black women survivors is needed. Transformative justice may serve as a tailored structural alternative solution for Black women survivors and their communities.

### Application of an afrocentric approach to transformative justice

Afrocentricity, brought to prominence in the United States by Molefi Kete Asante, is a paradigm defined as an approach to thought and behavior that centers African interests, values, and perspectives [28, 29]. This paradigm affirms and celebrates the right of people of African descent to pursue self-determination, which calls for the development of Afrocentric ontologies, epistemologies, and methodologies [30]. Key principles that guide Afrocentric thought and behavior include the promotion of unity, self-determination, collective responsibility, cooperative economics, collective purpose, creativity, and faith [31]. These tenets that prioritize collectiveness encompass a set of values that can help build and strengthen Black communities, and may be especially useful to consider as a framework for opposing structural racism that is historically and modernly embedded within social and legal services [32]. Drawing on an Afrocentric approach, transformative justice can lend itself to the seven major principles of the Afrocentric paradigm as part of a holistic response that resists the external systems that oppress Black individuals and embraces cultural heritage, collective empowerment, and cohesion. Findings from synthesis of qualitative research aimed at understanding strategies for Black women IPV survivors to overcome IPV suggest that Black women IPV survivors may prefer IPV-related services that are Afrocentric when available [33]. Further, transformative justice has been practiced and enhanced using Afrocentric principles to address non-IPV-related harm and has been shown to be a method to address intersecting injustices among Black individuals and transform the person who caused harm and their community [24]. When practiced within Black communities, transformative justice may help provide a tailored approach to IPV prevention and intervention that incorporates and aligns with Afrocentricity, and more effectively promotes accountability among people who cause harm and acquisition of justice among survivors than traditional criminal legal practices.

### Current study

Previous research has examined structural- and community-level predictors of police reporting among IPV and sexual violence survivors in the United States. This body of scholarship suggests that racism and sexism interact to produce inequitable police responses, ultimately leading to underreporting of IPV to police [34]. Moreover, research that has examined IPV and sexual violence survivors’ justice goals finds that survivors’ goals align closely with restorative justice practices, another survivor-centered alternative to the criminal legal response that prioritizes harm repair [35]. Finally, a study that used nationally representative data to examine how state-level restorative justice polices impact perceived mental health among women IPV survivors found that state restorative justice policies may be less effective in improving mental health outcomes among Black women IPV survivors compared to white IPV survivors [36]. Notably, however, samples in these studies were not specific to Black women IPV survivors, and little is known about justice preferences among Black women IPV survivors.

Because of the ways in which the criminal legal system fails Black women IPV survivors in particular, research on Black women IPV survivors’ perceptions of alternative forms of justice is needed [15]. It is crucial to understand Black women IPV survivors’ interactions with the police that respond to IPV and their preferences for justice to understand how to effectively apply, tailor, and transform the justice response to better serve Black women IPV survivors. This study uses intersectional and Afrocentric lenses to address this gap. We conducted a qualitative research study to understand Black women IPV survivors’ experiences with police interactions and their accountability preferences for IPV with the goal of understanding how to tailor the justice approach to IPV for Black women. We sought to answer the following questions: (1) What are Black women IPV survivors’ experiences interacting with police for IPV? (2) What are Black women IPV survivors’ preferences for justice and accountability for IPV?

## Methods

### Sample

Participants were from the Physiological Reactions to Women’s Experiences of Stress Study (PROWESS), an explanatory sequential mixed methods study (QUANT -> QUAL) conducted from November 2020 – April 2022 designed to understand experiences of stress among Black women IPV survivors. Participants for this study were recruited nationally using Instagram and Facebook social media platforms and Craigslist. Women were deemed eligible if they met the following criteria: (1) self-identified as a Black or African American cisgender female; (2) between 18 and 44 years old; (3) reported experiencing at least one incident of physical or sexual IPV with a male partner within the past year; and (4) engaged in sexual activity with a male partner in the past 6 months. Participants were screened for eligibility via telephone by a research team member or through a self-administered online survey. Informed consent was obtained from 55 participants who completed baseline and 1-month follow-up surveys. In the qualitative phase of the study, 15 of these participants were interviewed after completing the follow-up survey. These participants were not further sampled based on particular inclusion/exclusion criteria; rather, all participants were invited to participate in an interview, and recruitment stopped once 15 participants agreed to be interviewed. The sample size of 15 was considered sufficient due to reaching thematic saturation since themes were recurring and supported that no new information was being identified. The interview participants provided oral informed consent. Detailed characteristics of the sample are provided in the Results section. For more details about the parent study, see [37].

### Interview guide

Using an intracategorical intersectionality approach [38], semi-structured in-depth interviews with 15 Black women were conducted from April 2020 to April 2022. During the interviews, participants were asked about perceptions of how the Coronavirus Disease 2019 (COVID-19) pandemic impacted health, finances and relationships, which has been published [37]. For this analysis, participants were specifically asked a subset of questions about experiences interacting with the police for IPV, perceptions of interacting with the police for IPV, perceptions of healing from IPV, and preferred modalities for resolving experiences of IPV (**Supplementary File 1**). Interviewers used probes about justice during the interview using questions like, “What does justice for IPV look like for you?” The data forming the basis of this analysis have not been included in any previous publications from this project. Interviews were conducted virtually using a Zoom interface without use of the video feature except to initially verify participant identity and lasted an average of 60 min. The interviews were audio-recorded and transcribed by Landmark, an external transcription service. Participants were remunerated $25 via a gift card. All study procedures were approved by Yale University (HIC#2000027381) Human Investigation Committee and Johns Hopkins Bloomberg School of Public Health (#13147) Institutional Review Board.

### Data analysis

The research team drew upon inductive qualitative analytic techniques from grounded theory [39]. In alignment with the goals of grounded theory, we sought to expand beyond what is already known to define a new area of knowledge grounded in Black women IPV survivors’ lived experiences. First, an interdisciplinary team consisting of five coders, which included the qualitative interviewer and Principal Investigators, open-coded two interviews. In this stage, codes were iteratively discussed, combined, and condensed to create a preliminary codebook. Through these iterative practices, the codebook was refined and finalized. Using the final codebook, the remaining transcripts were coded by two coders, and any questions or inconsistencies that arose during this time were discussed by the research team and settled by the Principal Investigators. Next, the coding team used axial coding [39] to understand codes in relation to one another and develop themes. A matrix was used to organize themes by participant, which facilitated a deeper understanding of the distribution of responses and salience of themes. Finally, narrative summaries were created and used throughout the analysis process to contextualize case examples derived from the matrix. Dedoose version 9.0.62 [40] was used to analyze data. Pseudonyms have been assigned to each participant to preserve anonymity.

Four criteria of trustworthiness were applied to the qualitative analysis: credibility, dependability, transferability, and confirmability [41]. Credibility and dependability were strengthened through sustained engagement with the data, including multiple transcript reviews, robust coding, and peer debriefings where interdisciplinary scholars, including Black women and women who have experienced IPV, provided qualitative guidance. Additionally, the first author conducted member-checking, which included meeting with the 7 of the 15 participants who agreed to participate, to review our findings with them to ensure results matched their experiences and/or their perceptions of participants’ experiences to strengthen credibility. These participants were remunerated $45 via a gift card. Through these meetings, we found that our results aligned with their experiences and/or perceptions, and there was no need to modify or re-interpret results based on participant feedback. Transferability was strengthened by interviewing key informants from the parent study of purposively sampled Black women IPV survivors and providing a rich description of the participants, study context, results, and discussion of limitations to aid in the assessment of the extent to which study findings may transfer to other contexts and populations. Lastly, confirmability was strengthened by providing a detailed description of the research process.

### Researcher reflexivity

The research team took a constructivist-inclined critical epistemological approach to the design and analysis of the study. The research team includes individuals who represent or identify with the intersections that are pertinent to our participants and the aims of this study. These intersections include race, gender, lived experiences of IPV, lived experiences of police interactions for IPV, and lived experiences of interacting with the criminal legal system for IPV. Initials were not used to elucidate specific intersectional identities of the researchers to protect them.

The research team aimed to achieve socially constructed truths that would be co-created in the research process. The team’s intersectional identities, abolitionist stance regarding IPV, and prior knowledge about racism, sexism, and patterns of IPV influenced the questions participants were asked and probed with during interviews.

In the analysis phase of the study, the team recognized how the data would be influenced and co-constructed when presented. We chose to allow the data to inform us and recognized that we would bring in our identities, stances, and prior knowledge on the topic as researchers while analyzing and interpreting the data. Additionally, in alignment with critical theory, an awareness of power was acknowledged when the data were interpreted because we aim to use findings to help dismantle inequities and power structures related to the justice and accountability process for Black women IPV survivors.

## Results

### Participant characteristics

Table 1 presents demographic characteristics of the sample. The average age of the sample was 29.7 (4.1). The majority of the women self-reported experiencing both physical and sexual IPV (66.7%). About half of the women reported having never interacted with the police for IPV (53.3%). Most of the women finished college or graduate school (93.3%), were employed (86.7%), and made an annual salary above $30,000 (80.0%). All the women self-identified as cisgender (100.0%), and most of the women identified as heterosexual (93.3%). Lastly, slightly less than half the sample identified as Protestant (46.7%).

**Table 1:** Characteristics of 15 Black Women Intimate Partner Violence (IPV) Survivors

	*N* (%)
Overall	15 (100.0)
Age, M (SD)	29.7 (4.1)
IPV type	
Physical IPV only	2 (13.3)
Sexual IPV only	3 (20.0)
Both physical and sexual IPV	10 (66.7)
Ever interacted with the police for IPV	
Yes	3 (20.0)
No	8 (53.3)
Highest completed education	
Some college	1 (6.7)
Finished college	9 (60.0)
Finished graduate school	5 (33.3)
Employment status	
Unemployed	2 (13.3)
Employed (full or part-time)	13 (86.7)
Gender identity	
Cisgender	15 (100.0)
Sexual orientation	
Heterosexual	14 (93.3)
Bisexual	1 (6.7)
Income status	
<$30,000	3 (20.0)
$30,000+	12 (80.0)
Religion	
Catholic	4 (26.7)
Baptist	1 (6.7)
Protestant	7 (46.7)
Christian, non-denominational	2 (13.3)
None	1 (6.7)

* 4 responses are missing from, “Ever interacted with the police for IPV”

### Themes

The section below describes women’s experiences and perceptions interacting with the police for IPV. We discuss how these experiences are influenced by racism and sexism and note that participants themselves interpreted their interactions as racist and/or sexist.

### Fear and distrust of the police

The most salient theme expressed by participants was fear and distrust of the police. Fear of engagement with the police was mainly driven by anticipated discrimination resulting from lived experiences of discrimination when interacting with police for both IPV-related incidents and non-IPV-related events. However, some women anticipated discrimination despite having no lived experiences of discrimination by the police or interacting with police for IPV.

Tiffany was a mother of one child and married to a Black male partner. Tiffany had experienced physical abuse by her partner but had never called the police during any physically abusive events. Because she and her partner were both Black, she feared that they both “might end up being hurt” if they interacted with the police. When contemplating if she would ever interact with police for IPV, Tiffany stated:

*Probably if I’m assured or I’m certain that whatever I’m going to report won’t turn against me. That’s the only time I can turn to the police. I’m always never comfortable interacting with them. I’m saying I don’t like interaction with the police because they have a tendency of discriminating among the Black people*,* so reporting some issue*,* you might end up being the victim.*

Tiffany felt uncomfortable interacting with police due to fear of discrimination and being further victimized by police when calling them for IPV.

Contrastingly, Jasmine’s fear of discrimination stemmed from her direct lived experience of interacting with the police for a robbery. Jasmine, who was dating a white male partner, endured racialized discrimination when she contacted the police after being robbed. During the response to the robbery, a white female police officer told her, “That’s what you guys do. I’m sure that was someone you know, that took it. Or you are literally the thief,” which made her feel bad about herself as a Black woman and hesitant to rely on the police for help with IPV. Jasmine described:

*Yeah*,* sometimes I’m scared that I could be reporting this case to a white man or a white woman and it would be like*,* that’s what you deserve*,* because I’ve gotten such response before. They were like*,* that’s what you deserve. You Black people are actually hypocrites. I’m sure he didn’t do anything to you*,* because my boyfriend was white.*

Based on her previous racist experience with police when robbed, Jasmine feared that interacting with a police officer who had a different racial identity than her, but a congruent racial identity with her abusive partner, would result in her differential treatment during the justice response for IPV.

Bria, whose partner was also a white male, also anticipated discrimination from the police as part of their response to IPV, and this anticipation stemmed from her lived experience interacting with the police for non-IPV-related events. During her childhood, Bria frequently watched white police officers enter her home to arrest her Black father. She was surveilled during childhood as a Black girl walking down the street. Specifically, as a Black girl, she would be questioned, which made her feel “bad, humiliated, [and] discriminated [against],” and forced her to wonder if she would be arrested. She expressed:

*From my childhood trauma*,* I didn’t believe policemen would come and arrest a white man. The disadvantage that I had is if you were a white man*,* it’s almost…I didn’t even think of calling them because I just thought even if they arrest them*,* he would come back home tomorrow.*

Bria experienced the devaluation of Black girlhood through racialized and gendered discrimination by police. From these experiences, she anticipated being punished and devalued as a Black woman by police if she sought help for IPV. Like Jasmine, Bria believed her partner would be privileged due to his white male status, subsequently released, and ultimately not held accountable for the harm he caused her.

Serena, a mother of three daughters and one son, experienced fear and distrust resulting from her lived experience of interacting with the police specifically for IPV, and she also experienced exacerbation of trauma as a result of police discrimination. During an episode of conflict, Serena’s Black male partner grabbed her after he had been drinking, which prompted Serena to call the police. However, Serena’s partner left before the police arrived. She described:

*It was more of a headache than to—just being that I just wanted him to leave. They put a warrant out for his arrest and then called [Child Protective Service (CPS) Agency]. It was just a lot. It makes you not want to call if something little happens or if you wanna get him out the house. It just put a bad taste in my mouth. I don’t ever wanna call the police and then I’m in trouble because of a situation happened in front of the kids. Then they have to call [CPS Agency] and I have to deal with that.*

Serena did not receive a survivor-centered response from the police. She contacted the police desiring that they remove her abusive partner from their home, not arrest him. However, the police disregarded her preferences and focused on arresting him regardless of her wishes. Moreover, police exacerbated the trauma of IPV by reporting Serena to CPS. This response not only failed to create a space for Serena to exert her agency, but further fueled her distrust in the police and created a new avenue through which she would have to interact with a discriminatory system (CPS).

Some participants felt such strong distrust and fear of the police that they only relied on them to respond to IPV as a last resort, or when it was a matter of survival. Alexandra, a mother of one child and married to her Black male partner, related:

*Yeah ‘cause we had a fight. It was very serious and I got injured on my face—I’m sorry*,* my right eye. It was a hit and I had an injury and also it became swollen. Because the violence was increasing day in day out*,* I decide to report it to the police […] I think I had no choice. I’d gone to the end of the limit*,* so I had no other avenue and at this time I was in pain. I had been injured*,* so I felt that that’s the only option because I was feeling so much hatred towards my husband.*

Alexandra relied on the police only once she experienced severe physical abuse and felt she had no other alternative.

### Justice preferences

Four themes emerged that described Black women IPV survivors’ preferences for a comprehensive approach to justice, accountability, and healing from IPV: resolution through dialogue, therapy and counseling, support through increased access to resources, and protection of children.

#### Resolution through dialogue

When discussing their justice preferences, most women wanted to find IPV resolution through dialogue in a non-judgmental space. Survivors expressed that they preferred that dialogue be survivor-centered, facilitated by family or community members, and occur with or without their partner. Some participants specified that solution-based dialogue specifically include Black women in their community or other IPV survivors as a method of support.

Lila experienced physical and economic IPV. Her preference for justice was to contact her sister because she wanted someone she trusted to listen to her. When thinking about an ideal justice response to IPV, Lila described:

*Justice is where if you’re the victim*,* you are listened to and action is taken against the person who commits that crime where you are not judged. You are not discriminated [against]. You are given the right opportunity to express yourself without fearing that you’re being intimidated in one way or another. I think they can only be resolved through where the victims when they get there*,* they sit down and discuss the issues and to find the solution to the issues that are causing the conflicts […]*.

Lila explained that discussing IPV with a goal of finding solutions with trusted individuals is a more compassionate and tailored way to resolve conflict than depending on the police process because the dialogue is “personal.” Like Lila, Chloe appreciated being able to talk to her family to resolve IPV conflict. Beyond her family, she found it helpful to speak with other Black women as part of the justice response. Chloe stated:

*Havin’ support*,* my family is very supportive. That’s one thing that’s really helped me*,* and then also takin’ advantage of certain opportunities that are given*,* and havin’ a community of different Black women that’s support ’cause they can finally understand what I’ve been through.*

Relatedly, Sydney felt that talking with other IPV survivors she could identify with was an important component of the justice response to IPV. Sydney was a Black woman IPV survivor who was “so afraid” of the police because of their differential treatment towards Black individuals. Sydney explained:

*I feel like they should just maybe create a special program for women where they are able—where they are comfortable*,* and they are their self—like talk about their self*,* talk about the experience without being judged*,* talk about things they have gone through without being judged and without the fear of being judged. That’s just it. That would really*,* really help. I feel like talking about it makes it better.*

Sydney felt that IPV survivors could understand each other’s experiences, which could create a non-stigmatizing environment to promote healing as part of the conceptualization of justice.

Makayla was a mother of two children who also chose not to interact with police for IPV because of their treatment of Black individuals. When thinking about who should play a role in holding the person who caused harm accountable, Makayla suggested:

*I think the community should have elders that could try and mediate and mentor people on how to resolve issues.*

Makayla thought that designating trusted mediators in the community who could speak with individuals involved in IPV could promote community safety, and that collective support would help hold her partner accountable for the harm he caused her.

#### Therapy and counseling services

Many women also mentioned therapy and counseling services as an important component of the justice response for Black women IPV survivors. Some women preferred that the survivor and person who caused harm receive therapy and counseling services separately. Others felt that receiving counseling together could address the underlying causes of IPV, and ultimately change their partner’s behaviors and attitudes towards violence against women.

On one occasion, Jasmine’s mother came by their house and witnessed Jasmine’s partner physically abuse her. When her mother feared for Jasmine’s life, she suggested that she and Jasmine call the police for help. However, Jasmine did not want to involve the police in holding her partner accountable. Jasmine explained:

*I love him a lot. So I don’t want to see him locked behind bars because I feel that he can actually get proper help. I feel that he could go to a rehab*,* see a therapist or something*,* and he would be better. So I just felt that taking him to the cops would be so extreme. He could just spend a year or six months there*,* and I can’t imagine seeing him in pain.*

Jasmine suggested that instead, therapy services might more effectively help her partner reduce his violent behavior and would be her preferred way of holding him accountable for IPV. Relatedly, Sydney believed that both the IPV survivor and person who caused harm could benefit from counseling that they received separately. Sydney said:

*Yeah*,* not just the victim*,* but also the person with the evil. They should be given some sort of counseling because you need to make them understand that you’re going out there raping females. What happens if somebody do this to your mother or your sister? They need to understand that women are women. We all feel the same pain. They need some sort of counseling to make them—they feel like it’s okay. It’s good. They need to see the dangers of this thing.*

Similarly, Alexandra also believed counseling for the IPV survivor and person who caused harm was an important part of the justice response for IPV. However, she preferred engaging in counseling services together. Alexandra shared:

*I went for counseling. We went together actually and we did some sessions and again*,* the family members have been very*,* very much supportive. They talk to us often. They want to know how we are faring on*,* they want to know what I’m doing*,* and out of that*,* I became open and anytime I have any issue*,* I’m able to communicate with them. Again I’ve chosen to accept myself to heal and also to enjoy life. I go out dancing*,* clubbing and also socializing with friends. Above all I think we have improved very much on communication.*

Alexandra felt that going through counseling with her partner helped improve their conflict resolution and communication skills as a couple.

#### Resource support

Participants discussed increased access to resources like housing, daycare and transportation as critical to achieving justice for IPV. Housing was mentioned most often. For example, Chloe was with a partner who damaged her house and attempted to end her life. She called the police and was encouraged to obtain a protective order after they arrested her partner. She elected not to go this route due to “fear for retaliation” from her partner. When thinking about her justice preferences, Chloe expressed that she wished she was offered more resources to help keep her safe above and beyond a protective order. For example, she explained that survivors need more financial support to relocate after their abusive partners get out of jail. She commented on how:

*There’s nothin’ real in place to keep us safe after [the abuser has gotten out of jail]. […] I was offered a relocation*,* but they would only give me the first month [of housing financial support]. I didn’t have job at the time. It’s like*,* “How am I gonna continue to pay if I relocate?” It’s just unrealistic to ask you to relocate*,* or maybe those abusers should just never get out*,* or they should be relocated. I don’t know*,* but I just feel like somethin’ should be in place to keep survivors safe in the aftermath ’cause nothin’ like that exists*,* and so*,* a lotta people are reoffended [on] and everything else.*

Unlike Chloe, Bria had sustainable housing support to help her hold her partner accountable. Bria called her partner’s mother and disclosed IPV to her after a night of physical abuse. In response, her partner’s mother sent Bria’s partner to rehabilitation services for three months. During this time, Bria was able stay in a house in a different state that was provided by her partner’s family for safety. Once her partner returned from rehabilitation, the couple returned to the house they shared, and gathered his mother and father, and Bria’s mother to discuss the abuse. They eventually developed a plan whereby the entire family would convene once a month to discuss Bria’s partner’s progress on abstaining from abuse. Monthly check-ins helped hold her partner accountable in the short term while working towards complete long-term cessation of abuse. Bria stated:

*He arranged that from his dad and his mom. If he doesn’t cooperate again and stuff*,* the house where I used to go*,* where I lived before he came back*,* it was a—what can I call it? A prelim*,* where I would go if he decided not to behave […] I was told the house is mine. You can go back if you want to. Feeling that support and we felt like his family was very serious on this.*

These quotes exemplify survivors’ perception of justice that moves beyond individual retribution, and toward processes that harness community resources to restore fairness and equity at larger societal levels.

#### Protection and prevention for children

For some women, having children to consider critically shaped their preferences for justice for IPV, which suggested that participants’ conceptualization of justice and accountability for IPV went beyond the survivor. Many women wanted to protect their children from police exposure and further wanted their children to be educated about IPV through various systems and the community to prevent intergenerational IPV.

Kiara was a twenty-six-year-old single mother of two sons. She witnessed IPV as a child and experienced IPV in her marriage. Her decision to divorce her abusive partner and not involve police in her IPV experiences was largely influenced by her desire to protect her children. When considering her justice preferences in relation to her children, Kiara stated:

*I never want my kids to see a policeman in our house. They are boys*,* and for some reasons*,* Black males*,* your interaction with the police is not so good most of the time*,* so first I never want them to see a policeman in our house or anybody unless he’s a family friend coming to say hi*,* but not in uniform. I don’t want them to fear a policeman or to take them as a bad person*,* and here’s the thing*,* this is their dad. For some reason you have to think he’s their dad*,* and I don’t want them to see him as a bad person*,* but I think those were bad decisions.*

Makayla also decided not to involve the police to hold her abusive partner accountable because she did not want to subject herself, her partner, or her child to police discrimination. She also did not want her children to continue witnessing IPV, so she separated from her partner. Makayla described her vision for a holistic justice response:

*I think the most important thing what I think is having children being raised well*,* being taught about violence*,* the consequences. If you bring up a morally upright society*,* definitely things will be easier. They should start from parents. Parents should be role models to their children. Again*,* in schools it should be taught also as part of the curriculum and also in social places like churches.*

Makayla felt that multiple avenues should be used to promote IPV prevention among Black males, and that this education should begin with youth. For her, justice is not isolated to the two individuals involved in IPV but involves expanding education to community organizations and focusing on preventing future and intergenerational IPV. Alexandra also felt that education was important for preventing IPV among Black male youth and expanded upon how the community can be collectively responsible in taking this on. Alexandra shared:

*In my opinion*,* I think the elders have a role to educate the young men and discourage violence*,* also give them better avenues to solve conflict.*

## Discussion

To our knowledge, this is one of the first studies to explore justice preferences among a sample composed exclusively of Black women IPV survivors. When asked about preferences for justice for IPV, survivors’ preferences went beyond accountability for the focal person who caused harm and centered on the collective responsibilities of external systems and communities, which can be embodied through transformative justice. Specifically, participants endorsed resolution through dialogue, including discussions with family, dialogue led by trusted community members, discussions with women who have similar identities such as survivors or those identifying as Black women, and dialogues involving the person who caused harm. They identified counseling and psychological services, occurring individually and/or together, as part of a holistic justice response. Participants described their preference for having access to resources as part of the justice response to IPV with housing support being the most widely noted preference. Lastly, Black women IPV survivors preferred that the justice response to IPV center community-based prevention of IPV for children through education.

Our findings contribute to a growing body of literature that suggests that interacting with police as a primary justice approach for addressing IPV may be ineffective for Black women IPV survivors. Participants in this study were hesitant to rely on the police due to feared discrimination. This finding is consistent with previous research that suggests that Black women anticipate discrimination due to previous direct and indirect negative experiences with police [34, 42, 43]. This perception was salient for Black women who were devalued by police during childhood. As an extension of the historically inequitable treatment exhibited by the criminal legal system towards Black women, young Black girls have also been disproportionately punished by the criminal legal system, both historically and modernly, and are often perceived and treated as adults [10, 44]. Similar to stereotypes used to justify violence against Black women, Black girls are often racially stereotyped as devoid of innocence and are therefore not perceived as capable of being victimized [10]. This stereotype persists into Black womanhood, which contributes to the (both feared and realized) delegitimization of Black women’s experiences with IPV victimization and a failure by police to hold the person who caused harm accountable [10].

Additionally, the race of a survivor’s partner influenced anticipated discrimination. Having a white male partner exacerbated anticipated discrimination and mistrust of the police, especially if this was concordant with police racial identity. Black women survivors in our sample believed the criminal legal system would protect their white male partners if they sought help for IPV from the police. This is likely because these partners are men who *have* historically and modernly benefitted from patriarchy and white supremacy [8, 25]. On the other hand, having a Black male partner evoked survivors’ need to protect their Black partners from harmful and racist police brutality and discrimination. These phenomena, as described by women in our sample, undermine Black women’s claims to survivorship, and perpetuate violence from multiple sources (e.g., partner and state) [10]. Black women survivors in our study recognized how they and their Black male partners already face punitive sanctions at the hands of multiple systems [45], and this recognition led to Black women survivors prioritizing the safety of their Black partner and community over the safety of themselves. These behaviors align with Afrocentric principles of collectivism [32] and are congruent with previous research that further describes such safety trade-offs among Black women IPV survivors [34]. Research suggests that this “lose-lose situation” may, in part, be remedied by engaging Black women’s communities in transforming justice praxis and social conditions to better serve Black women IPV survivors [10, 44].

We also found that women who had prior experience interacting with the police for IPV experienced a non-survivor-centered justice and accountability response, which exacerbated existing trauma and ultimately made them regret relying on the police for help. Moreover, we found that survivors worried about their own safety and their children’s safety in addition to fearing that their abuse would be minimized by the police. This finding is consistent with research that documents instances of police contacting CPS, resulting in survivors losing custody of their children, and research documenting incarceration of the person who caused harm, despite the survivor’s wishes to avoid arrest [43, 46–49]. The widespread fear and distrust of the police led women in this sample to rely on police for help only if IPV was severe or life-threatening, and they felt there was no alternative. Black women IPV survivors should be able to seek help promptly, without feeling compelled to wait until the abuse is severe, and the resulting help-seeking response should be supportive rather than harmful, enabling them to access help from traditional avenues. These findings reflect the intersecting forms of violence (e.g., partner and state) that Black women IPV survivors undergo and the need for alternative justice approaches, particularly separate from the criminal legal system.

When thinking about IPV justice preferences, Black women IPV survivors in our sample preferred that both they and their partners undergo therapy and counseling, individually and/or together, to foster a holistic accountability process. While not all partners were Black men in this study, many Black women partner with Black men, and it is important to acknowledge that there may be socio-cultural barriers for Black men to receive these services [50]. These socio-cultural barriers include historical trauma, stigma, mistrust, cost, lack of mental health education, and access to services [51]. These socio-cultural barriers are further complicated by Black masculinity which does not socialize Black boys and men to be receptive to mental health services and psychotherapy [51, 52]. Therefore, there may be some cultural disconnect between Black women IPV survivors’ recommendations for their partners, and Black men who caused harm’s willingness to engage in these recommendations. Transformative justice practiced in Black communities may be used to help shift Black men’s perceptions of therapy and counseling services and leverage resources to connect Black women IPV survivors and their partners to therapy services in a survivor-centered approach to IPV justice.

Black women IPV survivors’ preference for housing security as a form of resource support powerfully illustrates the necessity of addressing basic needs and providing tangible resources as part of a holistic justice response for Black women experiencing IPV. This finding aligns with previous research highlighting the critical importance of housing stability for Black women IPV survivors [53–55]. Currently, IPV resources are heavily geared towards policing, criminal legal responses, and temporary shelter, rather than helping survivors remain in their communities and meet their daily needs [15, 53]. One important implication that can be drawn from this finding is the need to transform larger societal systems to be more equitable for these women. Communities can lead transformation by leveraging resources to help Black women IPV survivors meet basic needs, thereby collectively supporting survivors in holding their partners accountable, achieving sustained safety, and exercising their agency.

Children who are exposed to interparental IPV are more likely to experience IPV themselves, a pattern referred to as the “intergenerational transmission of violence” [56–58]. Survivors in this sample desired collective community responsibility to educate children about IPV and healthy relationships to help disrupt this intergenerational pattern and promote IPV prevention broadly. Sites for education and prevention that participants described included community schools and churches. These findings support the assertion that Black women IPV survivors do not see their ideal justice response as punitive, but as holistic. Survivors see their ideal justice response as one that does not rely on the state, but rather involves a collective, community-based approach that centers on prevention and intervention. As such, community members like educators, church leaders, and advocates should be involved in the transformative justice process for Black women IPV survivors and their children to promote holistic justice.

Transformative justice has been a longstanding practice in Black communities as an alternative approach to addressing IPV and providing options outside of the criminal legal system [24]. Given that practices that align with transformative justice are preferred by Black women IPV survivors, future research should focus on how to implement and use transformative justice for IPV to uplift Black communities and amplify their voices. However, research and guidance on implementing transformative justice for Black women IPV survivors and their communities remains scant. Kim et al. [59] conducted a study among nine domestic violence and sexual assault organizations to understand barriers and facilitators of implementing a transformative justice model approach into provider organizations and found that aligning organizational values with transformative justice principles, prioritizing training, and incorporating services for the person who caused harm were among methods to facilitate implementation of an intervention model aligned with transformative justice into community-based organizations. This study can provide valuable insights for implementing transformative justice in Black communities. However, it is important to acknowledge that while this study included organizations serving intersectional groups such as Asian and Latinx cisgender women, immigrant, and refugee populations, it did not include organizations primarily focused on serving Black women. Given the historical and social context for Black women and their communities, it is crucial to prioritize the unique experiences and preferences of the focal Black community to ensure the transformative justice approach implemented is tailored to meet their specific needs. Relatedly, future research should use community-based participatory research (CBPR) methods to facilitate the co-creation, co-design, and co-development of transformative justice with researchers [60]. CBPR is well-aligned with intersectionality’s principles of reflecting community realities and priorities, fostering equitable partnerships, and interpreting research findings within community contexts, which can be used to facilitate the dissemination and translation of findings to drive transformational social change for Black women IPV survivors and their communities [61]. Moreover, recent research has explored incorporating transformative justice praxis into domestic violence intervention programs [62]. While integrating transformative justice principles may bolster a survivor-centered approach of domestic violence intervention programs, caution is warranted when integrating these principles into formal services linked to the criminal legal system. This is crucial to avoid undermining the core foundation of the transformative justice movement as separate from the criminal legal system.

This study has limitations that warrant consideration. Our sample was comprised primarily of middle-class Black women who had attained at least some college education. Therefore, these findings may not be transferrable to other populations of Black women IPV survivors, such as women who are living at or below the poverty line or who are more socioeconomically marginalized. Further, these findings are likely not transferrable outside of the United States because the nature of justice responses may differ across contexts. We did not have geographical location information such as urbanicity of participants’ setting available to us to reflect on. Future research should include this important information since it may influence how Black women IPV survivors perceive the police. Since our sample consists of survivors from various communities across the United States, we believe that findings reflect diverse experiences and perspectives. Next, since these interviews were conducted between 2020 and 2022, it is possible that women experienced changes to IPV reporting due to the ongoing COVID-19 pandemic. We broadly inquired about Black women IPV survivors’ experiences with police but did not specifically focus on their encounters during the pandemic. Therefore, we cannot distinguish between their experiences before and during the pandemic. However, we did ask multiple questions about how the pandemic impacted their experiences of IPV. Here, COVID-19 pandemic-specific coercive control impacted their IPV experiences during this time [37].

## Conclusions

Overall, interacting with police as part of the criminal legal response was counterproductive for Black Women IPV survivors in our study, and Black women IPV survivors desired alternative forms of justice and accountability for IPV that align with a transformative justice approach. Transformative justice provides a unique opportunity for Black women IPV survivors and their communities to challenge interlocking systems of power such as racism and sexism that are deeply embedded in the workings of the criminal legal system and perpetuate harm within Black communities. Therefore, transformative justice may serve as a useful tool to promote equity and center Black women IPV survivors and their communities in an alternative justice response for IPV.

## Electronic supplementary material

Below is the link to the electronic supplementary material.

Supplementary Material 1

## Data Availability

The qualitative data generated and/or analyzed during the study are not publicly available because they contain information that could compromise participant privacy and/or consent. The corresponding author can be contacted for follow-up questions and/or concerns.

## Abbreviations

IPV: Intimate partner violence
CPS: Child protective services
CBPR: Community-based participatory research
COVID-19: Coronavirus Disease 2019
