# Supplementary material for: Moving towards transformative justice for black women survivors of intimate partner violence: an intersectional qualitative study
Source: BMC Public Health. 2024 Oct 8;24:2730. doi: 10.1186/s12889-024-20244-y (PMC11459893; doi:10.1186/s12889-024-20244-y)
Supplement: Supplementary file 1 — Supplementary Material 1 [file 12889_2024_20244_MOESM1_ESM.docx]

**Supplementary Table 1. Sample questions from the interview guide**

| - Can you tell me about a time when you interacted with the police because of conflicts with your romantic partner? |
| --- |
| - What would impact your willingness as a Black woman to interact with the police because of conflicts with your romantic partner? |
| - We can imagine that experiencing conflicts in your relationship is stressful. What would help you heal? |
| - Who do you think is best to help resolve experiences of conflict with a romantic partner? |
| - What are ways that your community could help resolve experiences of conflict with a romantic partner? |
| - What are ways state laws or policies could help resolve experiences of conflict with a romantic partner? |
